# Value of amniotic fluid homocysteine assay in prenatal diagnosis of combined methylmalonic acidemia and homocystinuria, cobalamin C type

**DOI:** 10.1186/s13023-021-01762-z

**Published:** 2021-03-10

**Authors:** Ting Chen, Lili Liang, Huiwen Zhang, Jun Ye, Wenjuan Qiu, Bing Xiao, Hong Zhu, Lei Wang, Feng Xu, Zhuwen Gong, Xuefan Gu, Lianshu Han

**Affiliations:** 1 Department of Pediatric Endocrinology and Genetic, Xinhua Hospital, Shanghai Institute for Pediatric Research, Shanghai Jiao Tong University School of Medicine, 1665 Kongjiang Road, Yangpu District, Shanghai, 200092 China; 2 Center for Prenatal Diagnosis, Xinhua Hospital, Shanghai Jiao Tong University School of Medicine, Shanghai, 200092 China

**Keywords:** Methylmalonic academia, Homocysteine, Prenatal diagnosis, *MMACHC* variant

## Abstract

**Background:**

Combined methylmalonic acidemia and homocystinuria, cobalamin C type (cblC defect) is the most common inborn error of cobalamin metabolism, and different approaches have been applied to its prenatal diagnosis. To evaluate the reliability of biochemical method for the prenatal diagnosis of cblC defect, we conducted a retrospective study of our 10-year experience at a single center.

**Methods:**

248 pregnancies whose probands were diagnosed as cblC defect were referred to our center for prenatal diagnosis from January 2010 to December 2019. Prenatal data of Hcy levels determined by enzymatic cycling assay, acylcarnitine analysis using liquid chromatography tandem mass spectrometry, organic acid analysis using gas chromatography mass spectrometry, and genetic analysis by direct sequencing of 248 at-risk fetuses were retrospectively reviewed.

**Results:**

For 2.0 and 16.0 μmol/L levels of Hcy AF samples, the relative errors were − 2.5% and 2.8%, respectively. The respective measurement uncertainties were 13.07% and 14.20%. For the 248 at-risk fetuses, 63 fetuses were affected and 185 fetuses were unaffected. Hcy level of 13.20 (6.62–43.30) μmol/L in 63 affected fetuses was significantly higher than that in 185 unaffected fetuses of 2.70 (0.00–5.80) μmol/L, and there was no overlap between the affected and unaffected groups. The diagnostic sensitivity and specificity of Hcy were 100% and 92.05%, respectively. The positive and negative predictive values of the combination of Hcy, propionylcarnitine (C3), ratio of C3 to acetylcarnitine (C2; C3/C2), methylmalonic acid (MMA), and methylcitric acid (MCA) were both 100%. Sixteen fetuses displayed inconclusive genetic results of *MMACHC* variants, in which seven fetuses were determined to be affected with elevated levels of Hcy, C3, C3/C2 and MMA, and their levels were 18.50 (6.70–43.30) μmol/L, 8.53(5.02–11.91) μmol/L, 0.77 (0.52–0.97), 8.96 (6.55–40.32) mmol/mol Cr, respectively. The remaining nine fetuses were considered unaffected based on a normal amniotic fluid metabolite profile.

**Conclusions:**

Hcy appears to be another characteristic biomarker for the prenatal diagnosis of cblC defect. The combination of Hcy assay with acylcarnitine and organic acid analysis is a fast, sensitive, and reliable prenatal diagnostic biochemical approach. This approach could overcome the challenge of the lack of genetic analysis for families with at-risk cblC defect fetuses.

**Supplementary Information:**

The online version contains supplementary material available at 10.1186/s13023-021-01762-z.

## Introduction

Combined methylmalonic academia and homocystinuria, cobalamin C type (cblC defect) is the most frequent genetic disorder of cobalamin metabolism [1]. The incidence of cblC defect ranges from 1:46,000 to 1:200,000 in European and American countries [2] and varies hugely from 1:3,220 to 1:21,488 in China [3–5].The cblC defect is caused by variants in the *MMACHC* gene located in chromosome region 1p34.1 [6]. This defect impairs the conversion of cobalamin to methylcobalamin and adenosylcobalamin, resulting in the accumulation of homocysteine (Hcy) and methylmalonic acid (MMA) [7]. Based on the age of onset, cblC defect has two distinct phenotypes. Patients with early-onset present clinical symptoms that include feeding difficulties, progressive developmental delay, and hypotonia within the first year of life. Patients with late-onset exhibit relatively milder clinical features, such as behavioral disturbances and progressive neurological symptoms, later in life [8]. Despite the early diagnosis and effective treatment, the outcome is not always favorable, especially in early-onset cblC defect [9–11]. Thus, a reliable method for the prenatal diagnosis of cblC defect is needed to inform decisions regarding continuation of pregnancies of cblC defect fetuses.

To some extent, the combination of acylcarnitine analysis (characteristic metabolite biomarkers of propionylcarnitine (C3), the ratio of C3 to acetylcarnitine (C2; C3/C2) by liquid chromatography tandem mass spectrometry (LC–MS/MS), organic acid analyses of characteristic metabolite biomarkers of MMA, and methylcitric acid (MCA) by gas chromatography mass spectrometry (GC–MS) is widely applied to the prenatal diagnosis of cblC defect [12, 13]. False positive and false negative results might also exist [14]. Thus, identifying another characteristic and sensitive biomarker to enhance the accuracy of metabolite analysis is desirable for the prenatal diagnosis of cblC defect. As cblC defect patients display a marked elevation of the plasma Hcy level [15, 16], an elevated Hcy level in amniotic fluid (AF) might serve as a characteristic metabolite biomarker for the prenatal diagnosis of cblC defect. Furthermore, the combination of Hcy assay with acylcarnitine and organic acid analysis in AF could be more reliable and precise for the prenatal diagnosis of cblC defect.

Here, we present our findings with 248 at-risk pregnancies whose probands were diagnosed as cblC defect in the prenatal diagnosis of cblC defect by metabolite analysis of AF supernatants with/without genetic analysis of amniocytes. The aim was to elucidate the value of AF Hcy assay in the prenatal diagnosis of cblC defect.

## Methods

### Families and probands

In this study, 226 families (248 pregnancies) in which the probands were diagnosed as cblC defect were referred to our center for prenatal diagnosis from January 2010 to December 2019. The probands were diagnosed based on the symptoms, metabolite results of elevated blood levels of C3, C3/C2, MMA and MCA, and/or with genetic analysis. Informed consent forms were signed by the parents or legal guardians of the study participants. This study was approved by the Ethics Committee of Xinhua Hospital (approval number XHEC-D-2020-131).

### AF sample

In each case, 30 mL sample of AF was collected at 16–20 weeks of gestation from the pregnant woman. Of the 30 mL, 10 mL was used for DNA extraction. The cell-free AF supernatant was used for metabolite analysis. For this analysis, 13 μL was used for Hcy determination. 2 mL was used for organic acids analysis. 3 μL was used for quantitative acylcarnitine analysis. The remaining 20 mL of AF was cultured for karyotyping analysis, with the cultured amniocytes also used as a back-up.

### Hcy determination of AF

Thirteen microliters of each cell-free AF sample was centrifuged at 3000 r/min for 5 min. The level of Hcy in AF was measured by an enzymatic cycling assay using the Hcy assay kit (Beijing Jiuqiang Biotechnology, Beijing, China) and following the manufacturer’s protocol using an automatic analyzer (Hitachi, Tokyo, Japan). To interpret the results of Hcy assay in AF, the degree of measurement uncertainty and relative error were investigated. A low level of 2.0 μmol/L and a high level of 16.0 μmol/L of Hcy AF samples were measured. Within-day imprecision was assessed by measuring six replicates of the two Hcy levels of AF samples, and values of co-efficient of variation (*CV*_*W*_) were calculated. Between-day imprecision of Hcy assay was assessed by daily analysis for 6 days of a single lot of the two Hcy levels of AF samples, and *CV*_*B*_ were calculated. Method bias was assessed by measuring the two standard Hcy levels of AF samples in every 3 days for 6 times, and *CV*_*Bias*_ were calculated. The inspection quality of Hcy assay was assessed by calculating the measurement uncertainty (*U* = $\sqrt{{{CV}_{W}}^{2}+{{CV}_{B}}^{2}+{{CV}_{Bias}}^{2}}\times \kappa$) with the coverage factor *κ* = 2.

### Acylcarnitine analysis of AF by LC–MS/MS

The level of acylcarnitine in each AF samples was analyzed using an API-4000 tandem mass spectrometer (Applied Biosystems, Foster City, CA, USA). Pretreatment of each AF sample and the LC–MS/MS operating conditions were previously reported [17]. Quantitative analysis of acylcarnitine was achieved using the ratio of the averaged ion intensity to that of the corresponding internal standards.

### Organic acid analysis by GC–MS in AF

Organic acids in AF were analyzed using a single quadrupole GCMS-QP 2010 device (Shimadzu, Kyoto, Japan). Pretreatment of AF sample and GC–MS operating conditions were previously reported [17]. Quantitative analysis of each organic acid was achieved using the relative peak area of each Q-ion to that of the corresponding internal standards.

### Direct *MMACHC* variant screening by Sanger sequencing

Genomic DNA was extracted from the cultured amniocytes using the DNA extraction kit (TIANGEN Biotech, Beijing, China) according to the manufacturer’s instruction. The conditions of the PCR reactions and analysis of DNA sequencing were previously described [18]. Nucleotide variations were identified using a reference sequence from Genbank (*MMACHC*: NM_015506). The novel variants were searched using the Mutalyzer website tool (https://mutalyzer.nl/) and the Human Gene Mutation Database.

### Diagnostic criteria

Biochemical analysis: The reference ranges of Hcy, C3, C3/C2, MMA and MCA were1.10–4.10 μmol/L, 0.30–4.00 μmol/L, 0.05–0.25, 0.00–1.00 mmol/mol Cr and 0.00–0.50 mmol/mol Cr, respectively. Fetuses with the above metabolites’ levels higher than the upper limit of reference ranges were suggested as cblC defect.

Genetic analysis: Fetuses harbored homozygous variants or compound heterozygous variants of *MMACHC* were diagnosed as cblC defect.

### Statistical analysis

Data analysis was performed using SPSS 24.0 (IBM, Chicago, Illinois). Data of detected metabolites were not normally distributed. The data are presented as median (range). The Mann–Whitney U test was applied to compare the difference of the levels of the metabolites between the affected and unaffected groups. A *p* value less than 0.05 was considered statistically significant with a 95% confidence interval.

## Results

The metabolite and genetic analyses involved 248 at-risk fetuses. The combination of these analyses identified 56 fetuses as affected, with 176 fetuses considered unaffected. Of the remaining 16 fetuses with unavailable causative variants in the probands, based on AF metabolites analysis alone, 7 fetuses were determined to be affected and diagnosed as cblC defect, and 9 fetuses were judged as unaffected.

### Biochemical analysis of AF metabolites

The results of AF metabolites inferred that 63 of the totals of 248 fetuses were affected, with the remaining 185 unaffected (Table 1).

**Table 1:** Measurement uncertainties of two levels of Hcy in amniotic fluid

True value of Hcy (μmol/L)	Measured median value of Hcy (μmol/L)	*CV*_*W*_ (%)	*CV*_*B*_ (%)	*CV*_*Bias*_ (%)	*U* (%)
2.00	1.95	2.13	4.45	4.29	13.07
16.00	16.45	1.61	3.90	5.71	14.20

*Hcy* homocysteine, *CV*_*W*_ within-day imprecision, *CV*_*B*_ between-day imprecision, *CV*_*Bias*_ method bias imprecision, *U* measurement uncertainty

### Hcy level in AF

**Fig. 1:**
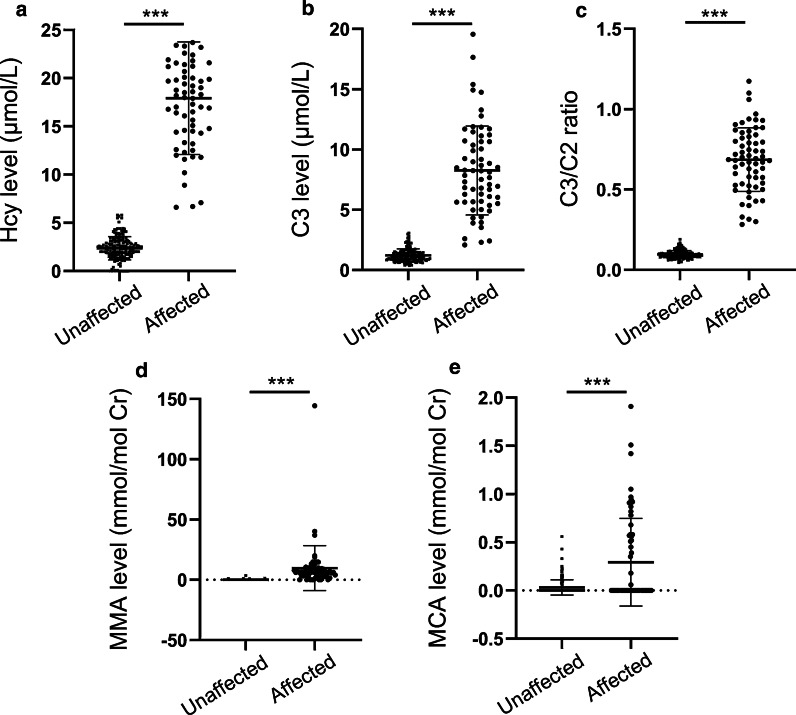
Scatter-plot showing the distribution of characteristic metabolite levels in affected and unaffected fetuses. **a** The distribution of Hcy levels between affected and unaffected fetuses; **b** the distribution of C3 levels between affected and unaffected fetuses; **c** the distribution of C3/C2 ratios between affected and unaffected fetuses; **d** the distribution of MMA levels between affected and unaffected fetuses; **e** the distribution of MCA levels between affected and unaffected fetuses. ***Significantly difference (*p* < 0.001) between the affected and unaffected groups

For the standard levels of 2.0 μmol/L and 16.0 μmol/L of Hcy AF samples, the relative errors were − 2.5% and 2.8%, respectively. The respective within-day imprecisions were 2.13% and 1.61%, the respective between-day imprecisions were 4.45% and 3.90%, and the respective method bias imprecisions were 4.29% and 5.71%. The measurement uncertainties of the selected two levels (2.0 and 16.0 μmol/L) of Hcy were 13.07% and 14.20%, respectively (Table 1). For the 185 unaffected fetuses, the median (range) level of Hcy was 2.70 μmol/L (0.00–5.80), which was significantly elevated in 63 affected fetuses with a median (range) level of 13.20 μmol/L (6.62–43.30) (*p* < 0.0001; Fig. 1). There was no overlap of the Hcy level between the affected and unaffected fetuses. All the individual levels of Hcy in 63 affected fetuses were higher than the defined reference range. Among the 185 unaffected fetuses, the Hcy levels in 15 fetuses were above the upper limit of the reference range.

### Acylcarnitine and organic acid levels in AF

As shown in Fig. 1, the median (range) levels of C3, C3/C2, MMA, and MCA in the AF of 185 unaffected fetuses were 1.08 (0.37–3.07) μmol/L, 0.09 (0.05–0.19), 0.00 (0.00–3.65) mmol/mol Cr, and 0.00 (0.00–0.56) mmol/mol Cr, respectively. These levels were notably increased in the 63 affected fetuses with the corresponding metabolite median (range) levels of 8.01 (2.09–19.58) μmol/L, 0.69 (0.28–1.17), 6.22 (0.00–144.40) mmol/mol Cr, and 0.00 (0.00–1.91) mmol/mol Cr, respectively (*p* < 0.0001). Among the 63 affected fetuses, the C3 levels in 7 fetuses, MMA levels in 8 fetuses, and MCA levels in 44 fetuses were within the reference range. Among the 185 unaffected fetuses, the MMA levels in 4 fetuses and the MCA level in one fetus were above the upper limit of the reference range.

### Genetic analysis of pathogenic variants in amniocyte DNA

Among the 248 at-risk fetuses, there were 232 fetuses with clear information concerning pathogenic variants in the probands and parents. For the 232 probands and 56 affected fetuses with clear *MMACHC* variants information, 59 cases harbored homozygous variants. The remaining 229 cases harbored compound heterozygous variants. Forty different variants were found. Of these, 9 variants were novel (see Additional file 1). The c.609G > A and c.658_660delAAG variants were the most common variants and accounted for 61.15% of disease alleles.

A total of 16 fetuses displayed inconclusive genetic results. Of these, 12 fetuses had only one causative mutation in the probands and there was a lack of available genetic information in the probands in 4 fetuses.

### Comparison of metabolite results and genetic results

Among the 232 fetuses with clear *MMACHC* variants information in the probands and parents, genetic and metabolite results in 169 fetuses were completely consistent. Twelve fetuses were affected and the remaining 157 were unaffected. For 63 fetuses, the metabolite results of Hcy, C3, C3/C2, MMA, and MCA were incompletely consistent with the genetic results (Table 2). Of these 63 fetuses, 44 were affected and 19 were unaffected. For the 44 affected fetuses, compared with the genetic results, inconsistent findings were evident for C3 of 3 fetuses, MMA of one fetus, MCA of 29 fetuses, and C3 and MCA of 4 fetuses. For the remaining 7 fetuses, the MMA and MCA results were inconsistent. For the 19 unaffected fetuses, inconsistencies with the genetic results were evident for the Hcy of 14 fetuses, MMA of 4 fetuses, and MCA of one fetus.

**Table 2:** Prenatal data of 63 fetuses with one or more metabolites results inconsistent with genetic results

No.	Variants of fetus (NM_015506)		Metabolites				
	Allele 1	Allele 2	Hcy (μmol/L)	C3 (μmol/L)	C3/C2	MMA (mmol/mol Cr)	MCA (mmol/mol Cr)
1	c.80A>G	c.658_660delAAG	13.32	**2.42**	0.30	1.55	0.51
2	c.568insT	c.467G>A	18.30	5.37	0.85	**0**	0.92
3	c.80A>G	c.481C>T	22.41	4.41	0.66	8.49	**0**
4	c.481C>T	Exon1 deletion	18.76	8.15	0.75	6.44	**0**
5	c.80A>G	c.609G>A	14.60	**3.96**	0.43	14.75	0.97
6	c.609G>A	c.656_658delAAG	13.20	10.06	0.74	5.58	**0.45**
7	c.394C>T	c.609G>A	12.50	6.95	0.63	**0**	**0**
8	c.609G>A	c.656_658delAAG	16.10	6.31	0.61	**0**	0.55
9	c.482G>A	c.609G>A	7.10	6.71	0.54	**0**	**0.06**
10	c.80A>G	c.609G>A	16.90	11.15	0.58	6.57	**0**
11	Exon1 deletion	c.599G>A	28.90	14.76	1.17	144.40	**0**
12	c.80A>G	c.658_660delAAG	15.10	**2.30**	0.33	3.64	**0**
13	c.455_457delCCC	c.658_660delAAG	17.00	11.73	0.63	**0**	**0**
14	c.609G>A	c.445_446delTG	21.40	9.19	0.83	13.31	**0**
15	c.428C>T	c.658_660delAAG	12.60	6.04	0.53	9.64	**0**
16	c.80A>G	c.609G>A	23.70	7.33	0.67	5.88	**0**
17	c.80A>G	c.609G>A	26.60	8.69	0.82	3.35	**0**
18	c.394C>T	c.445_446delTG	27.80	8.24	0.79	9.35	**0**
19	c.445_446delTG	c.609G>A	16.50	15.41	0.93	1.79	**0**
20	c.609G>A	c.658_660delAAG	12.00	5.67	0.41	7.53	**0**
21	c.57_58insT	c.609G>A	18.60	11.85	0.94	6.13	**0**
22	c.609G>A	Carry the same paternal allele as the proband	20.10	14.95	0.92	6.13	**0**
23	c.217C>T	c.609G>A	14.30	**3.90**	0.50	6.24	**0**
24	c.609G>A	c.626_627delTG	14.40	12.79	0.70	7.28	**0**
25	c.445_446delTG	c.609G>A	23.40	10.73	0.90	12.96	**0**
26	c.609G>A	c.658_660delAGA	17.00	10.67	0.74	7.78	**0**
27	c.394C>T	c.656_658delAGA	10.20	6.69	0.40	**0**	**0**
28	c.609G>A	c.658_660delAGA	17.30	7.53	0.52	6.82	**0**
29	c.80A>G	c.609G>A	21.80	6.11	0.49	7.09	**0**
30	c.567dupT	c.99delA	19.70	19.58	0.89	6.04	**0**
31	c.609G>A	c.658_660delAAG	19.80	10.38	0.58	3.85	**0**
32	c.80A>G	c.609G>A	11.80	5.70	0.32	**0**	**0**
33	c.567dupT	c.609G>A	21.20	11.71	0.64	10.64	**0**
34	c.482G>A	c.482G>A	6.62	**2.61**	0.43	5.95	**0.39**
35	c.445_446delTG	c.609G>A	19.19	**3.54**	0.68	3.2	1.51
36	c.482G>A	c.482G>A	8.90	**2.09**	0.28	4.01	**0**
37	c.609G>A	c.609G>A	20.00	17.65	0.79	**0**	**0**
38	c.609G>A	c.609G>A	19.00	9.91	0.60	5.71	**0**
39	c.609G>A	c.658_660delAAG	22.60	6.81	0.69	2.37	**0**
40	c.609G>A	c.609G>A	16.40	7.87	0.80	5.07	**0**
41	c.609G>A	c.609G>A	19.67	9.25	0.80	14.95	**0**
42	c.609G>A	c.609G>A	17.90	6.85	0.69	37.16	**0**
43	c.609G>A	c.658_660delAAG	21.60	11.48	0.87	6.22	**0**
44	c.609G>A	c.658_660delAAG	20.70	13.30	0.86	6.65	**0**
45	c.217C>T	–	**4.10**	0.72	0.10	0	0
46	c.658_660delAAG	–	**4.40**	1.46	0.15	0	0.16
47	c.658_660delAAG	–	**4.37**	0.40	0.09	0.91	0.08
48	c.609G>A	–	**4.42**	1.70	0.13	0	0
49	c.656_658delAGA	–	**5.70**	1.05	0.09	0	0.2
50	c.658_660delAAG	–	**5.80**	0.73	0.07	0	0
51	c.658_660delAAG	–	**4.30**	0.52	0.08	0	0
52	–	–	**5.50**	1.47	0.09	0	0
53	c.80A>G	–	**5.50**	0.97	0.16	0	0
54	c.609G>A	–	**5.10**	2.67	0.13	0	0
55	–	–	**4.30**	1.32	0.14	0	0
56	–	–	**4.10**	3.07	0.16	0	0
57	c.609G>A	–	**4.20**	2.99	0.08	0	0
58	–	–	**5.80**	1.15	0.11	0	0
59	–	–	1.55	0.55	0.08	**1.44**	0
60	–	–	2.60	0.60	0.11	**1.06**	0
61	c.609G>A	–	2.80	2.62	0.11	**1.16**	0
62	c.658_660delAAG	–	2.20	0.97	0.05	**3.65**	0
63	c.609G>A	–	2.00	1.02	0.09	0	**0.56**
	Reference range		1.10–4.10	0.30–4.00	0.05–0.25	0.00–1.00	0.00–0.50

Bold parts, metabolite results are inconsistent with the genetic results

*Hcy* homocysteine, *C3* propionylcarnitine, *C2* acetylcarnitine, *MMA* methylmalonic acid, *MCA* methylcitric acid

Among the 16 fetuses with inconclusive genetic results, 7 were inferred to be affected. Of these 7 fetuses, the levels of all metabolites were elevated in 3 fetuses. In the remaining 4 fetuses, the levels of Hcy, C3, C3/C2, and MMA were elevated, while the MCA level was not. For the other 9 unaffected fetuses, the levels of all the metabolites were normal in 8, with only the Hcy level being increased in the remaining fetus. These 9 fetuses showed a normal phenotype at postnatal follow-up (see Additional file 2).

For the 232 fetuses with information of the pathogenic variants in the probands and parents, comparison of the metabolite results with genetic results revealed the sensitivity and specificity of Hcy were 100% and 92.05%, respectively. The positive predictive values of Hcy, C3, C3/C2, MMA, and MCA were 80%, 100%, 100%, 92.31%, and 94.12%, respectively. The respective negative predictive values were 100%, 96.17%, 100%, 95.56%, and 81.39%. The positive and negative predictive values of the combination of those metabolites were both 100%.

## Discussion

The cblC defect is the most common subtype of vitamin B_12_ metabolism [19]. Even with rapid diagnosis and effective treatment, the long-term outcome remains unsatisfactory, especially in patients with early onset, because of severe neurological sequelae [11]. The families of these patients suffer heavy economic burdens. Thus, prenatal diagnosis can provide important information in the decision about the pregnancy involving a fetus with the cblC defect, which could further reduce the social and family pressures from this disease.

For the prenatal diagnosis of cblC defect, metabolites can be measured and monitored in AF [20]. In a notable example, Ji et al. [14] reported that metabolite analysis of acylcarnitines by LC–MS/MS and organic acids by GC–MS in AF could serve as rapid and reliable methods for the prenatal diagnosis of methylmalonic acidemia. However, their data of C3, C3/C2, MMA, and MCA sensitivity (95.1%, 100%, 100%, and 82.9%, respectively) and specificity (98.7%, 99.3%, 97.4%, and 96.7%, respectively) indicated the possibility of false positive results for the analyses of acylcarnitines and organic acids. Therefore, we aimed to find another effective biomarker to enhance the accuracy of metabolite analysis for prenatal diagnosis of the cblC defect.

Plasma total Hcy is recommended as an biomarker in the guideline for the diagnosis of cblC defect patients [15]. The Hcy assay of AF was performed in 9 at-risk fetuses for the prenatal diagnosis of cblC defect [21]. However, published reports concerning Hcy assay in AF for the prenatal diagnosis of cblC defect were all case reports or small series [21, 22]. Thus, it has been difficult to verify the reliability of the Hcy assay in the prenatal diagnosis of cblC defect. In this context, we retrospectively reviewed 248 at-risk fetuses with prenatal diagnostic data collected over a 10-year period to analyze the value of Hcy in the prenatal diagnosis of cblC defect. We first assessed the imprecisions of Hcy assay, the *CV*_*W*_, *CV*_*B*_ and *CV*_*Bias*_ values were all less than 10% [23] and proved that Hcy assay in AF was stable. Moreover, to interpret the results of Hcy assay in AF, the relative errors of the selected two levels (2.0 and 16.0 μmol/L) of Hcy were − 2.5% and 2.8%, respectively. The respective measurement uncertainties were 13.07% and 14.2%. Besides, we observed no overlap of the Hcy level between the affected and unaffected fetuses. By contrast, C3/C2 displayed no overlap, while the levels of C3, MMA, and MCA all overlapped between the affected and unaffected fetuses.

Among the 232 fetuses with information concerning pathogenic variants in the probands and parents, results of the Hcy, C3, C3/C2, MMA, and MCA metabolites showed discrepancies in 63 fetuses. Consequently, a biochemical prenatal diagnosis of cblC defect in these fetuses could be uncertain based on any one of the metabolites. Nevertheless, we noted that Hcy levels and C3/C2 were consistent with genetic results in all affected fetuses, while in all unaffected fetuses, C3 and C3/C2 levels were completely consistent with the genetic results. Thus, by taking advantage of the 100% sensitivity, Hcy could help to decrease the false negative rate and obtain a more accurate biochemical prenatal diagnosis in the affected fetuses. However, in contrast with C3, C3/C2, MMA, and MCA, Hcy showed the lowest specificity of 92.05%, which might be associated with the selection of the reference range. Generally, the setting of the reference range is calculated by taking twice the standard deviation from the mean or considering certain percentiles of the normal population [24].And the reference ranges of biomarkers for clinical diagnosis are commonly set as the 5th to 95th percentile of the normal population [25]. Thus, in our study, the reference range of Hcy was determined to be the 5th to 95th percentile of the Hcy values of 109 fetuses who were not at-risk for cblC defect. However, a previous report also suggested that a reference range based exclusively on normal population might result in many false positive results. Thus, the reference range might need adjustment in light of the overlap between the normal and disorder populations [26]. Therefore, depending on the increasing data of AF Hcy and the prenatal diagnosis of cblC defect, the reference range of prenatal Hcy level needs to be adjusted in the future to improve the low specificity of Hcy for the prenatal diagnosis of cblC defect. It is also worth noting that a total of 3 affected fetuses harboring the homozygous or heterozygous variant of c.482G > A displayed lower levels of Hcy (ranging from 6.62 to 8.9 μmol/L) compared to the other affected fetuses. The connection between the c.482G > A variant and Hcy level warrants a further investigation.

Genetic analysis is generally recognized as the gold standard for prenatal diagnosis [27, 28]. However, despite its accuracy, genetic analysis depends entirely on the complete genetic information of the proband and parents. For some at-risk fetuses without more than one causative variant found in the probands or when genetic analysis was not performed, this can hinder a precise prenatal diagnosis by genetic analysis alone. Presently, there were 12 fetuses without more than one causative variant and 4 fetuses whose genetic analysis were not performed. For these 16 fetuses, prenatal diagnosis was made depending on biochemical analysis alone. In a condition where the metabolite results were inconsistent, like the 5 fetuses in our study (Additional file 2), previous data indicates a preference for the more sensitive and specific biomarkers in the prenatal diagnosis [29]. Following this rule, among the 5 fetuses in the present study, 4 were diagnosed as the cblC defect. The one fetus diagnosed as unaffected showed a normal phenotype at the postnatal follow-up. Therefore, in this situation in which the genetic analysis alone did not permit a precise prenatal diagnosis, biochemical analysis for the supernatant of AF would provide fast and reliable results using a small amount of AF sample. The data could help families in making decisions concerning the pregnancies. This advantage was also observed in our previous reports on prenatal metabolite analysis in methylmalonic acidemia and glutaricacidemia-I [14, 30]. More importantly, the positive and negative predictive values for each metabolite in solo and in combination revealed that the combination of metabolites presented a greater reliability. Therefore, despite the accuracy of genetic analysis for prenatal diagnosis of cblC defect, the combination biochemical analysis of Hcy, C3, C3/C2, MMA, and MCA in AF appears to be valuable in the prenatal diagnosis of families for whom genetic results are not available.

## Conclusions

The Hcy characteristic metabolite appears to be a sensitive biomarker for the prenatal diagnosis of cblC defect. The combination of the Hcy assay with acylcarnitine and organic acid analysis offers a fast, sensitive, and reliable prenatal diagnostic biochemical approach, which could overcome the challenge of the lack of genetic data for families at-risk of cblC defect fetuses.

## Supplementary Information

**Additional file 1.** *MMACHC* gene variants for 232 probands and 56 affected fetuses. Description of data: The cDNA change, amino acid change, exon, effect and frequency of different *MMACHC* gene variants identified from 232 probands and 56 affected fetuses with cblC defect.

**Additional file 2.** Prenatal diagnostic results of 16 fetuses with inconclusive genetic results. Description of data: The metabolites’ results and inconclusive genetic results of 16 fetuses.

## Data Availability

The datasets supporting the conclusions of this article are included within the article and its additional files.

## Abbreviations

cblC defect: Combined methylmalonic acidemia and homocystinuria, cobalamin C type
AF: Amniotic fluid
LC–MS/MS: Liquid chromatography tandem mass spectrometry
GC-MS: Gas chromatography mass spectrometry
Hcy: Homocysteine
MMA: Methylmalonic acid
C3: Propionylcarnitine
C2: Acetylcarnitine
MCA: Methylcitric acid
