# Supplementary material for: Value of amniotic fluid homocysteine assay in prenatal diagnosis of combined methylmalonic acidemia and homocystinuria, cobalamin C type
Source: Orphanet J Rare Dis. 2021 Mar 10;16:125. doi: 10.1186/s13023-021-01762-z (PMC7945211; doi:10.1186/s13023-021-01762-z)
Supplement: Supplementary file 1 — Additional file 1. MMACHC gene variants for 232 probands and 56 affected fetuses. Description of data: The cDNA change, amino acid change, exon, effect and frequency of different MMACHC gene variants identified from 232 probands and 56 affected fetuses with cblC defect. [file 13023_2021_1762_MOESM1_ESM.docx]

Additional file 1 *MMACHC* gene variants for 232 probands and 56 affected fetuses

| No. | cDNA change | Amino acid change | Exon | Effect | Frequency (%) |
| --- | --- | --- | --- | --- | --- |
| 1 | c.1A>G | p.Met1Val | 1 | missense variant | 0.35 |
| 2 | **c.57dupT** | p.Gly20Trpfs^*^14 | 1 | frameshift variant | 0.35 |
| 3 | c.80A>G | p.Gln27Arg | 1 | missense variant | 8.71 |
| 4 | c.81+1G>A | IVS1+1G>A | intron1 | splicing variant | 0.17 |
| 5 | c.99delA | p.Glu33Aspfs^*^43 | 2 | frameshift variant | 0.35 |
| 6 | c.178dupG | p.Asp60Glyfs^*^18 | 2 | frameshift variant | 0.17 |
| 7 | c.217C>T | p.Arg73Ter | 2 | nonsense variant | 3.14 |
| 8 | c.228_231delTGAC | p.Asp77Glnfs^*^22 | 2 | frameshift variant | 0.70 |
| 9 | c.271dupA | p.Arg91Lysfs^*^14 | 2 | frameshift variant | 0.17 |
| 10 | c.276+1G>A | IVS2+1G>A | intron 2 | splicing variant | 0.17 |
| 11 | c.315C>G | p.Tyr105Ter | 3 | nonsense variant | 1.57 |
| 12 | c.331C>T | p.Arg111Ter | 3 | nonsense variant | 0.17 |
| 13 | c.365A>T | p.His121Leu | 3 | missense variant | 0.17 |
| 14 | c.394C>T | p.Arg132Ter | 3 | nonsense variant | 2.96 |
| 15 | c.395_397delGAC | p.Arg132del | 3 | deletion variant | 0.17 |
| 16 | c.427C>T | p.Gln143Ter | 3 | nonsense variant | 0.70 |
| 17 | c.445_446delTG | p.Cys149Hisfs^*^32 | 4 | frameshift variant | 3.31 |
| 18 | c.455_457delCCC | p.Pro152del | 4 | deletion variant | 0.35 |
| 19 | c.465_467delGGG | p.Gly155del | 4 | deletion variant | 0.52 |
| 20 | c.467G>A | p.Gly156Ala | 4 | missense variant | 0.52 |
| 21 | c.481C>T | p.Arg161Ter | 4 | nonsense variant | 1.05 |
| 22 | c.482G>A | p.Arg161Gln | 4 | missense variant | 3.14 |
| 23 | **c.531delA** | p.Lys178Asnfs^*^32 | 4 | frameshift variant | 0.17 |
| 24 | c.561_572del | p.Asp188_Ala191del | 4 | deletion variant | 0.17 |
| 25 | c.567dupT | p.Ile190Tyrfs^*^13 | 4 | frameshift variant | 5.40 |
| 26 | c.567_568insCCTT | p.Ile190Profs^*^14 | 4 | frameshift variant | 0.17 |
| 27 | **c.578T**>**G** | p.Leu193Arg | 4 | missense variant | 0.17 |
| 28 | c.599G>A | p.Trp200Ter | 4 | nonsense variant | 0.35 |
| 29 | **c.606**_**614del** | p.Trp203_Tyr205del | 4 | deletion variant | 0.17 |
| 30 | c.609G>A | p.Trp203Ter | 4 | nonsense variant | 45.99 |
| 31 | c.615C>A | p.Tyr205Ter | 4 | nonsense variant | 0.17 |
| 32 | **c.620A**>**T** | p.Asp207Val | 4 | missense variant | 0.17 |
| 33 | **c.626**_**627delTG** | p.Val209Aspfs^*^35 | 4 | frameshift variant | 0.52 |
| 34 | c.626dupT | p.Thr210Aspfs^*^35 | 4 | frameshift variant | 0.17 |
| 35 | **c.634delC** | p.Gln212Argfs^*^79 | 4 | frameshift variant | 0.17 |
| 36 | **c.653dupA** | p.Gln219Alafs^*^26 | 4 | frameshift variant | 0.17 |
| 37 | c.658_660delAAG | p.Lys220del | 4 | deletion variant | 15.16 |
| 38 | c.666C>A | p.Tyr222Ter | 4 | nonsense variant | 0.17 |
| 39 | Exon1 del |  | 1 | deletion variant | 1.57 |
| 40 | **Exon2 del** |  | 2 | deletion variant | 0.17 |

Novel variants identified in this study are shown in bold type.
