# Supplementary material for: Value of amniotic fluid homocysteine assay in prenatal diagnosis of combined methylmalonic acidemia and homocystinuria, cobalamin C type
Source: Orphanet J Rare Dis. 2021 Mar 10;16:125. doi: 10.1186/s13023-021-01762-z (PMC7945211; doi:10.1186/s13023-021-01762-z)
Supplement: Supplementary file 2 — Additional file 2. Prenatal diagnostic results of 16 fetuses with inconclusive genetic results. Description of data: The metabolites’ results and inconclusive genetic results of 16 fetuses. [file 13023_2021_1762_MOESM2_ESM.docx]

Additional file 2 Prenatal diagnostic results of 16 fetuses with inconclusive genetic results

| Prenatal diagnosis | Variants of proband | | Variants of fetus | | | Fetus metabolites | | | | |
| --- | --- | --- | --- | --- | --- | --- | --- | --- | --- | --- |
|  | Allele 1 | Allele 2 | Allele 1 | Allele 2 | Hcy  (μmol/L) | | C3  (μmol/L) | C3/C2 | MMA  (mmol/mol Cr) | MCA  (mmol/mol Cr) |
| Affected | untested | untested | untested | untested | 43.30 | | 11.91 | 0.97 | 7.95 | 0.57 |
| Affected | untested | untested | untested | untested | 21.58 | | 5.02 | 0.77 | 40.32 | 1.42 |
| Affected | c.394C>T | - | c.394C>T | - | 11.6 | | 8.53 | 0.66 | 9.16 | 0.18 |
| Affected | c.609G>A | - | c.609G>A | - | 12.3 | | 5.64 | 0.52 | 9.97 | 0.56 |
| Affected | c.656_658delAAG | - | c.656_658delAAG | - | 18.5 | | 7.11 | 0.71 | 8.79 | 0 |
| Affected | untested | untested | untested | untested | 6.7 | | 11.23 | 0.89 | 6.55 | 0 |
| Affected | c.217C>T | - | c.217C>T | - | 19.4 | | 8.99 | 0.93 | 8.96 | 0 |
| Unaffected | c.609G>A | - | - | - | 3.90 | | 1.14 | 0.09 | 0 | 0 |
| Unaffected | c.658_660delAAG | - | c.658_660delAAG | - | 1.20 | | 1.50 | 0.10 | 0 | 0 |
| Unaffected | c.609G>A | - | c.609G>A | - | 2.65 | | 1.15 | 0.15 | 0 | 0.22 |
| Unaffected | c.609G>A | - | - | - | 1.79 | | 0.93 | 0.10 | 0 | 0 |
| Unaffected | c.658_660delAAG | - | - | - | 1.99 | | 1.39 | 0.11 | 0 | 0.16 |
| Unaffected | untested | untested | untested | untested | 2.40 | | 1.37 | 0.12 | 0 | 0 |
| Unaffected | c.609G>A | - | - | - | 2.50 | | 0.85 | 0.11 | 0 | 0 |
| Unaffected | c.609G>A | - | c.609G>A | - | 3.20 | | 1.48 | 0.09 | 0 | 0 |
| Unaffected | c.658_660delAAG | - | - | - | 4.40 | | 1.42 | 0.07 | 0 | 0 |
| Reference ranges | | | | | 1.10-4.10 | | 0.30-4.00 | 0.05-0.25 | 0.00-1.00 | 0.00-0.50 |

Abbreviations: Hcy, homocysteine; C3, propionylcarnitine; C2, acetylcarnitine; MMA, methylmalonic acid; MCA, methylcitrate acid
